# Simulation and Study of Power Quality Issues in a Fixed Speed Wind Farm Substation

**DOI:** 10.1155/2015/367540

**Published:** 2015-04-08

**Authors:** T. Magesh, C. Chellamuthu

**Affiliations:** Department of EEE, R.M.K Engineering College, Kavaraipettai, Tamil Nadu 601 206, India

## Abstract

Power quality issues associated with the fixed speed wind farm substation located at Coimbatore district are investigated as the wind generators are tripping frequently. The investigations are carried out using two power quality analyzers, Fluke 435 and Dranetz PX5.8, with one of them connected at group control breaker of the 110 kV feeder and the other at the selected 0.69 kV generator busbar during the period of maximum power generation. From the analysis of the recorded data it is found that sag, swell, and transients are the major events which are responsible for the tripping of the generators. In the present study, simulation models for wind, turbine, shaft, pitch mechanism, induction generator, and grid are developed using DIgSILENT. Using the turbine characteristics, a two-dimensional lookup table is designed to generate a reference pitch angle necessary to simulate the power curve of the passive stall controlled wind turbine. Various scenarios and their effects on the performance of the wind farm are studied and validated with the recorded data and waveforms. The simulation model will be useful for the designers for planning and development of the wind farm before implementation.

## 1. Introduction

With the development of wind turbine technology, large scale wind farms of hundreds of MW are developed in many countries. These modern wind farms connected to the power grid will effectively reduce the requirement on the fossil fuel based conventional power generation [1]. Wind energy is commercially and operationally the most viable renewable energy resource in the world. In India, Tamil Nadu state ranks first with an installed capacity of 4287 MW of wind power out of available power of 8451 MW [2]. The integration of wind power generation into the existing power grid presents technical challenges and it requires consideration of voltage regulation, stability, and power quality. The power quality issues are the major problems that occur in the power grid and their causes are not properly identified [3]. Power quality indices have to be maintained in a power system according to the standards of EN50160 and IEC 61400-21. These standards define the measurement and assessment of the power quality characteristics of the grid-connected wind generation and are widely accepted by the wind turbine manufacturers and utilities. The important factors to be considered in power quality measurements are the active power, reactive power, variation in voltage, flicker, harmonics, and transient response due to switching operation [4, 5].

The various power quality issues are measured by various researchers at various states in India. Based on measurements and analyses, preliminary recommendations for integration of wind turbines in weak grids have been formulated by them [6]. However, the simulation model is not developed and only the preliminary study is executed based on the measured data. The operation of wind turbine is a major parameter contributing towards the power quality of the connected grid. Depending on the grid strength and the type of wind turbine used, different power quality problem may develop. The power fluctuation mainly due to wind power variation may cause the flickering effect in the system [7, 8]. They focused only on the flickering due to tower shadow and wind gradient but not the other issues such as sag, swell, interruption, and transients. The fixed speed wind farm is considered, by researchers, for the analysis, because it is simple, robust, reliable, and cost effective when compared to the variable speed wind farm [9]. The fluctuation in the wind speed is transmitted as the fluctuations in the mechanical power which leads to the variation in electrical power on the grid causing voltage fluctuation. The simulation model of wind electric system is developed using DIgSILENT software to analyze the power quality [10–14]. The pitch control mechanism is used to keep the rated output power by adjusting the pitch angle with the wind speed when it is above its rated value. The various optimal controllers are used for the pitch control mechanism for improving their performance [15]. However, the response of the controller is slow and the power fluctuation is high. The fixed speed wind electric generator imports reactive power from the grid leading to poor power factor [16]. When the generated active power of an induction generator varies due to changes in wind speed, the absorbed reactive power and terminal voltage of the generator also fluctuate. A proper compensator is required to minimize the reactive power absorbed from the grid. A STATCOM based control scheme has been developed in DIgSILENT [17] for improving the power quality of the grid [18].

In the proposed work, a detailed study of various recorded power quality issues and the simulation study of 3.6 MW wind farm with substation is carried out.  Section 2 discusses the layout of the substation and the rating of different components. The recorded power quality events are analysed and categorized in Section 3.  Section 4 discusses the mathematical model of fixed speed wind electric system (FSWES) and the simulation model of FSWES developed in DIgSILENT software. In Section 5, the results of simulation of various events are compared with those measured in the wind farm to validate the simulation model.  Section 6 describes a remedial measure to mitigate the effect of sag. Finally the conclusion is discussed in Section 7.

## 2. Wind Farm under Study

**Figure 1:**
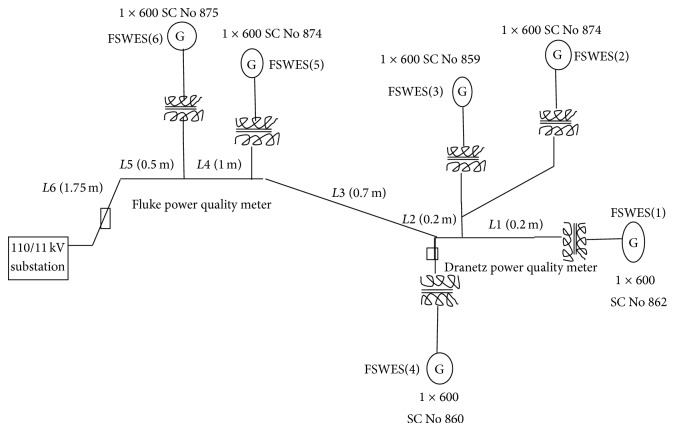
Layout of windfarm.

A wind farm in Coimbatore district, Tamil Nadu, is selected for power quality measurement and simulation studies as there is frequent disconnection of wind generators when maximum wind energy is available. The layout diagram of the wind farm giving the location of wind generator units from the substation is shown in Figure 1.

The wind farm consists of six units of fixed speed passive stall controlled wind turbines. Each turbine is connected to a 600 kW, 690 V squirrel cage induction generator. The total capacity of the wind farm is 3.6 MW. The generator is directly connected to the LV side of 0.85 MVA, 690 V/11 kV transformer. The reactive power compensation is provided by using a switching capacitor. The length of the cable connecting various FSWES to the substation is shown in Table 1. The wind farm substation is connected to the grid through 5 MVA, 11 kV/110 kV power transformer.

## 3. Measured Data at the Substation

In order to evaluate the power quality of the system conforming to IEC 61400-21 Standard [4], the data loggers Drantez PX5.8 and Fluke 435 are installed at a selected point of the system. The power quality disturbances are recorded for nearly twenty-nine days between the months of July and August 2011 as the wind flow was at the maximum. The primary aim is to analyze the various power quality issues faced by the substation connected to the wind farm.

**Figure 2:**
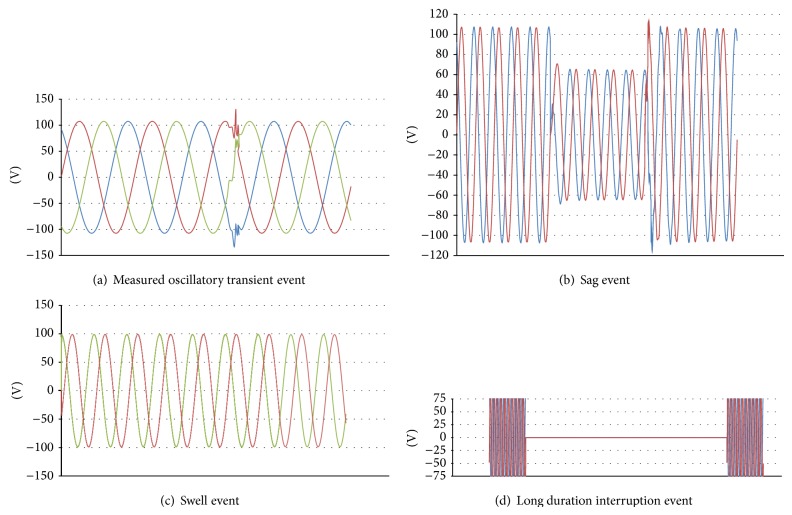
Waveform of various recorded events.

Several events related to the voltage quality are recorded by the instrument and they are categorized as transients, sag, swell, and interruptions. Of all the events catalogued, there are thirty-four impulsive transients. The authentic causes that initiate such issues are found to be switching of capacitors, switching of reactors, lightning, and utility fault clearing. The other disturbances are twenty sag events, three swell events, and two interruption events. The sample recorded waveforms for various events are shown in Figures 2(a)–2(d).

## 4. Modelling of Fixed Speed Wind Electric System (FSWES)

**Figure 3:**
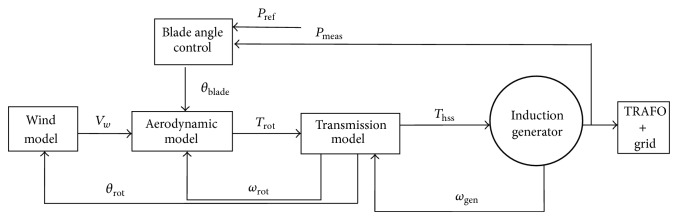
General block diagram of FSWES.

Each component of the FSWES is modelled in a dedicated power system simulation tool, DIgSILENT14.1.6 Power Factory. In this work, the model of the grid and the electrical components are selected from the standard library of the DIgSILENT. The models of mechanical components such as aerodynamic and control parts of the wind turbines and the wind flow are developed by using dynamic simulation language (DSL) [19]. The different blocks for the simulation of FSWES are shown in Figure 3. The major blocks are wind, aerodynamics, transmission, induction generator, and pitch angle control.

### 4.1. Wind Flow Model

Wind is an intermittent source of energy which is an outcome of air flow among the areas of varying pressure. Measuring the changes in the pressure facilitates the prediction of the wind speed in a particular region. The model of wind speed consists of four components as given in the following [12]:

(1) $$\begin{array}{c} V_{w}\left(t\right)=V_{\text{wa}}+V_{\text{wr}}\left(t\right)+V_{\text{wg}}\left(t\right)+V_{\text{wt}}\left(t\right). \end{array}$$

In this equation, *V*_*w*_(*t*) represents the wind speed, *V*_wa_ is the average value of wind speed, *V*_wr_(*t*) is the ramp component, *V*_wg_(*t*) is the gust component, and *V*_wt_(*t*) is the turbulence component. All these data are expressed in “meter per second” and the time is in “seconds.”

### 4.2. Aerodynamic Model

The aerodynamic power “*P*_wind_” developed by the turbine with rotor radius “*R*” at a wind speed “*V*_*w*_” and air density *ρ* is expressed as

(2) $$\begin{array}{c} P_{\text{wind}}=\frac{1}{2}\rho \pi R^{2}{V_{w}}^{3}C_{p}\left(\lambda ,\beta \right). \end{array}$$

The power coefficient *C*_*p*_ depends on the blade angle *β* and the tip speed ratio *λ* which is expressed as

(3) $$\begin{array}{c} \lambda =\frac{R\omega }{V_{w}}. \end{array}$$

The power coefficient *C*_*p*_ can be expressed as

(4) $$\begin{array}{c} C_{p}=\frac{\left(0.44-0.0167\beta \right)\sin\left(\pi \left(\lambda -2\right)\right)}{\left(13-0.3\beta \right)}-0.0018\left(\lambda -2\right)\beta . \end{array}$$

### 4.3. Shaft Model

A two-mass representation has been selected for a low speed shaft and is characterized by

(5) $$\begin{array}{c} \frac{d\omega_{\text{wt}}}{dt}=\frac{T_{\text{wr}}-K_{s}\gamma }{2H_{\text{wr}}},\\ \frac{d\omega_{m}}{dt}=\frac{K_{s}\gamma -T_{e}}{2H_{m}},\\ \frac{d\gamma }{dt}=2\pi f\left(\omega_{\text{wr}}-\omega_{m}\right). \end{array}$$

In the above equations, *f* is the nominal grid frequency, *T* is the torque, *γ* is the angular displacement of the two ends of the shaft, *ω* is electrical angular frequency, *H* is the inertia constant, and *K*_*s*_ is the stiffness of the shaft. The subscripts wr, *m*, and *e* stand for turbine rotor and mechanical and electrical variables, respectively. All values are in per unit except *K*_*s*_ and *f* which are in radians and hertz, respectively.

### 4.4. Model of Pitch Controller

**Figure 4:**
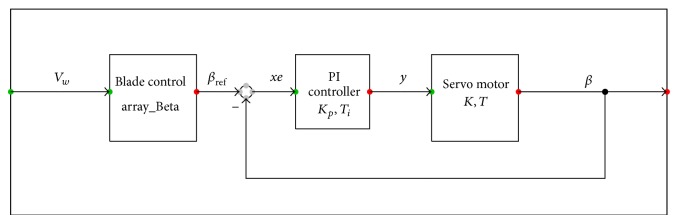
Model for pitch control.

During the times of high wind velocity, the rotor speed has to be maintained at about its rated value. This is implemented by employing servo mechanisms in each blade. The model of pitch controller as shown in Figure 4 consists of a reference block, PI block, and servo motor block. Instead of taking the generator speed, turbine speed, and generator power as the references, change in the wind speed is sensed. Therefore the response time of pitch control is improved. The blade control block is a lookup table which gives reference beta for a given wind speed so that the turbine runs at the rated speed above the nominal wind speed.

### 4.5. Induction Generator Model

Induction generator is modeled in the *d*-*q* stator reference frame in DIgSILENT as given in the following [19]:

(6) $$\begin{array}{c} u_{ds}=-R_{s}i_{ds}-\omega_{s}\psi_{qs}+\frac{d\psi_{ds}^{}}{dt},\\ u_{qs}=-R_{s}i_{qs}+\omega_{s}\psi_{ds}+\frac{d\psi_{qs}}{dt},\\ u_{dr}=0=-R_{r}i_{dr}-s\omega_{s}\psi_{qr}+\frac{d\psi_{dr}}{dt},\\ u_{qr}=0=-R_{r}i_{qr}+s\omega_{s}\psi_{dr}+\frac{d\psi_{qr}}{dt}. \end{array}$$

In the equations, *s* is the slip, *u* is the voltage, *i* is the current, *R* is the resistance, and *ψ* is the flux linkage. All quantities are in per unit. The subscripts *d* and *q* stand for direct and quadrature components, respectively, and the subscripts *r* and *s* are for rotor and stator, respectively.

The flux linkages in the above equations are calculated as given in

(7) $$\begin{array}{c} \psi_{ds}=-\left(L_{s\sigma }+L_{m}\right)i_{ds}-L_{m}i_{dr},\\ \psi_{qs}=-\left(L_{s\sigma }+L_{m}\right)i_{qs}-L_{m}i_{qr},\\ \psi_{dr}=-\left(L_{r\sigma }+L_{m}\right)i_{dr}-L_{m}i_{ds},\\ \psi_{qr}=-\left(L_{r\sigma }+L_{m}\right)i_{qr}-L_{m}i_{qs}, \end{array}$$

where *L* is the inductance. The indices *m*, *r*, and *σ* stand for mutual, rotor, and leakage quantities, respectively.

### 4.6. Simulation Model of FSWES in DIgSILENT

**Figure 5:**
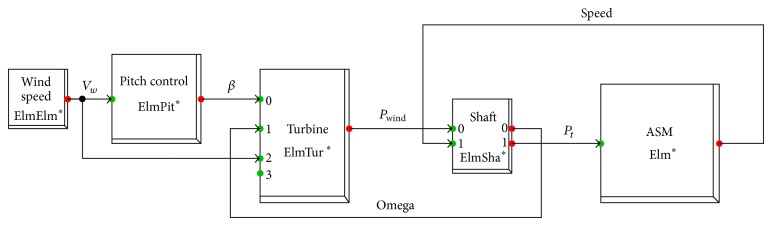
FSWES model in DIgSILENT.

**Figure 6:**
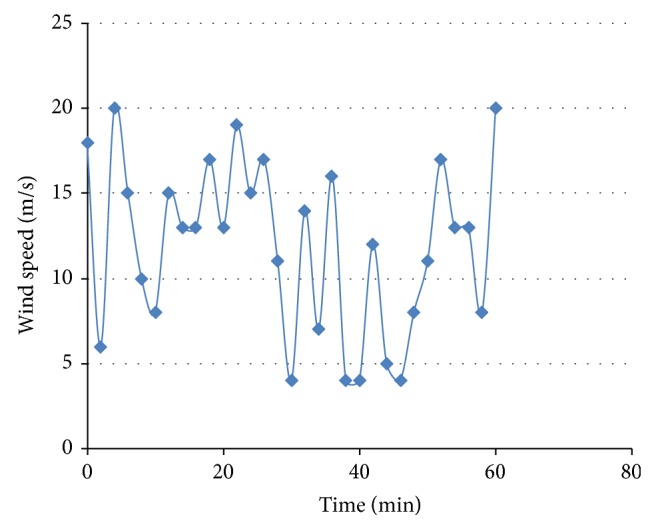
Time series model for wind.

The equations of the various functional blocks are implemented in DIgSILENT to realize a software model for FSWES as shown in Figure 5. The wind power block is modelled as a time series with 10 min intervals based on the recorded data in the site and is shown in Figure 6.

The aerodynamic turbine block is modelled using (2) to (4) with the help of DSL programming. The block has *V*_*w*_, *β*, and omega as the input and *P*_wind_ as the output power.

**Figure 7:**
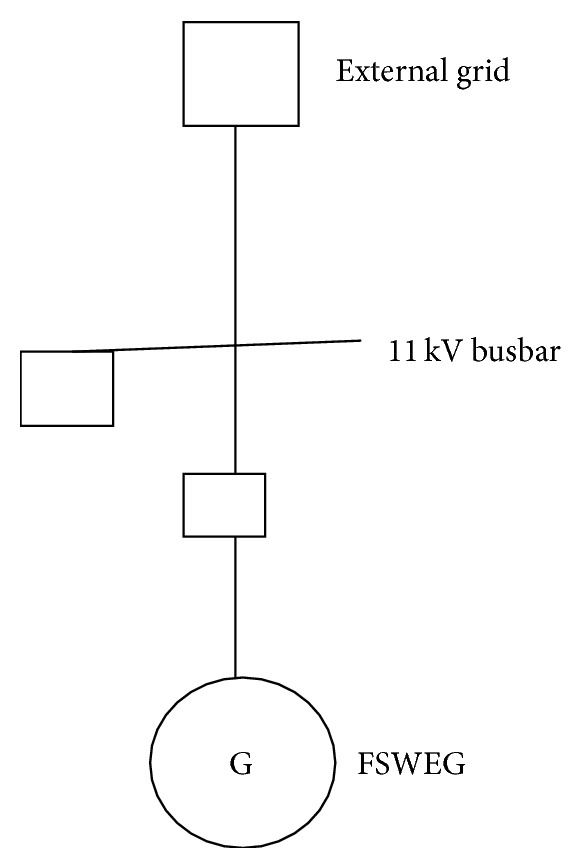
Representation of FSWEG in DIgSILENT.

The two-mass-shaft model has been simulated using (5). The mechanical turbine power is converted from lower shaft speed into higher shaft speed. The inputs to the shaft block are *P*_wind_ and generator's speed whereas the *P*_*t*_ and omega are the output variables. The power output *P*_*t*_ is connected to the mechanical input terminal of the built-in library block of asynchronous machine. The pitch controller block takes the input as *V*_*w*_ and calculates appropriate value of beta to keep the power output constant when the wind speed is higher than the rated value. The FSWES model shown in Figure 5 is combined with the unit transformer to represent them as FSWEG which is used as a simulation model in the wind farm as given in Figure 7 [20].

### 4.7. Simulation Model of Wind Farm

**Figure 8:**
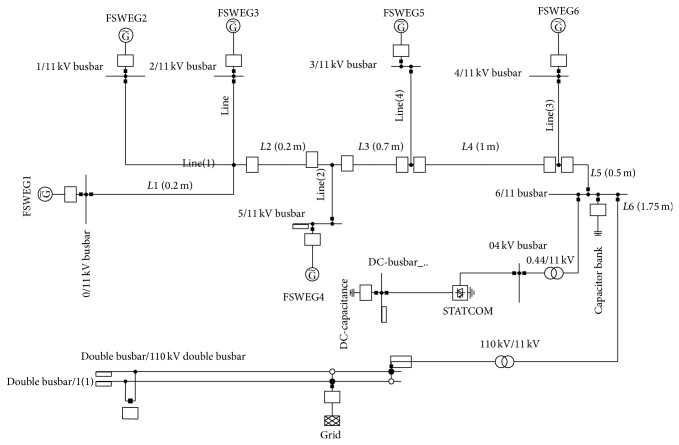
Simulation model of wind farm.

The layout of the substation is implemented using the model of FSWEG and built-in models of power system components like busbar, transformer, cable, converter, and soft starter as shown in Figure 8. The simulation model of wind farm consists of six FSWEGs of similar rating and they are connected to 11 kV busbar through underground cable. A STATCOM model, developed for reactive power compensation, is connected to 11 kV bus through 0.44 kV/11 kV transformer. The 110 kV double busbar linking the grid is connected to 11 kV substation bus through power transformer.

## 5. Simulation Scenarios and Results

This section furnishes the simulation details for studying the consequences of various events that are likely to occur in the wind farm at any given period. The RMS simulation is run using simulation software, DIgSILENT. Further, the simulation results are compared with the results recorded in the wind farm to validate the proposed simulation model.

### 5.1. Load Flow Studies of the Wind Farm

**Figure 9:**
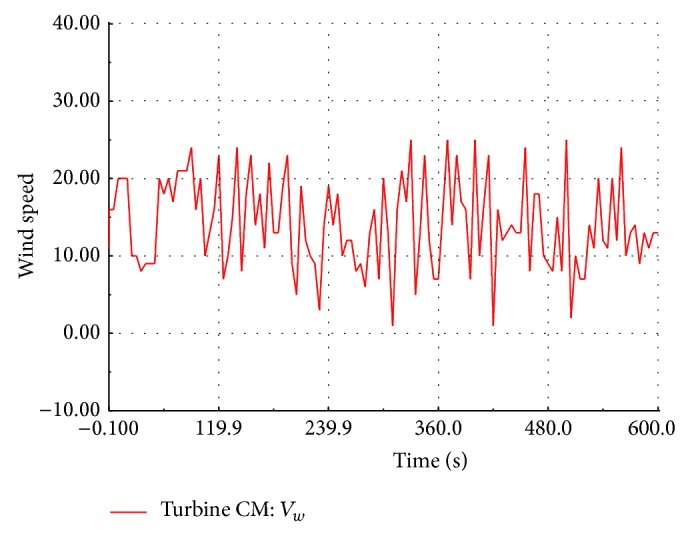
Simulated wind pattern.

**Figure 10:**
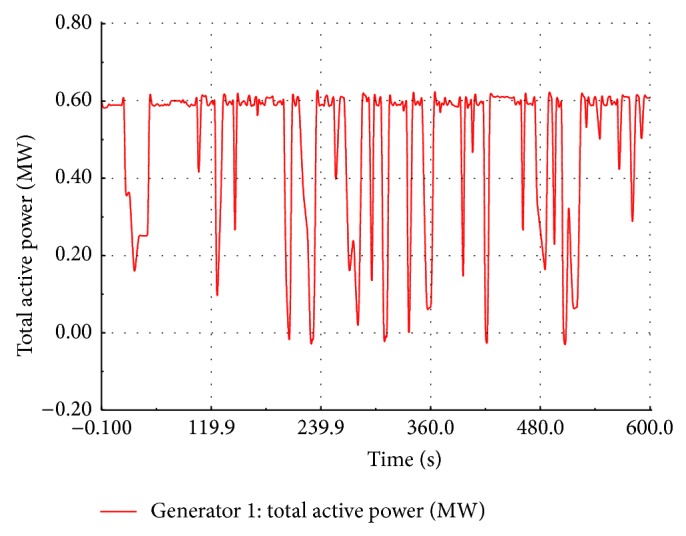
Simulated real power at FSWEG1.

**Figure 11:**
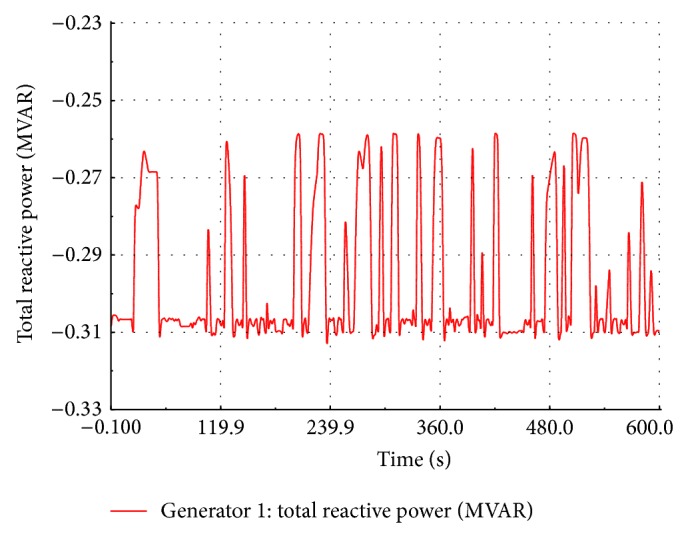
Simulated reactive power at FSWEG1.

An RMS transient simulation is run for ten minutes to evaluate the power flow in different section of the wind farm. The wind speed is assumed to be the same value for all the FSWEGs in the wind farm and it varies between cut-in and cut-out values (3 m/s to 20 m/s) as shown in Figure 9. When the wind speed is above the rated value 12 m/s, the pitch control mechanism becomes active to maintain a constant real power at the generator terminal. When the wind speed is below the rated value, the pitch control mechanism becomes inactive. The real power variation with respect to wind speed is shown in Figure 10. It is observed that the power is maintained at 0.6 MW above the rated wind speed whereas the power varies proportionally to the cube of the wind speed below its rated value. The reactive power absorption from the grid also changes with the wind speed as shown in Figure 11. The absorbed reactive power by the individual wind generator varies from 0.20 MVAR to 0.31 MVAR. Under full load condition, the total reactive power absorption from the grid including the underground cable is 1.97 MVAR and it is compensated by a 2 MVAR shunt capacitor connected at 11 kV substation bus.

### 5.2. Short Circuit Studies of the Wind Farm

**Figure 12:**
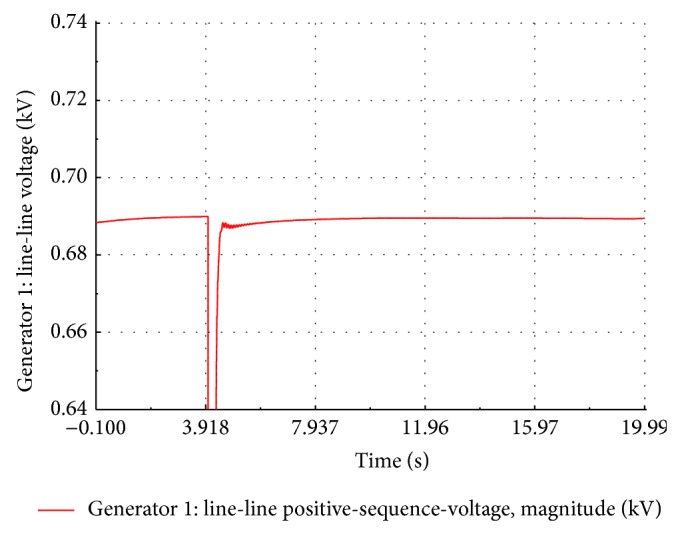
Simulated voltage dips at FSWEG1 busbar.

**Figure 13:**
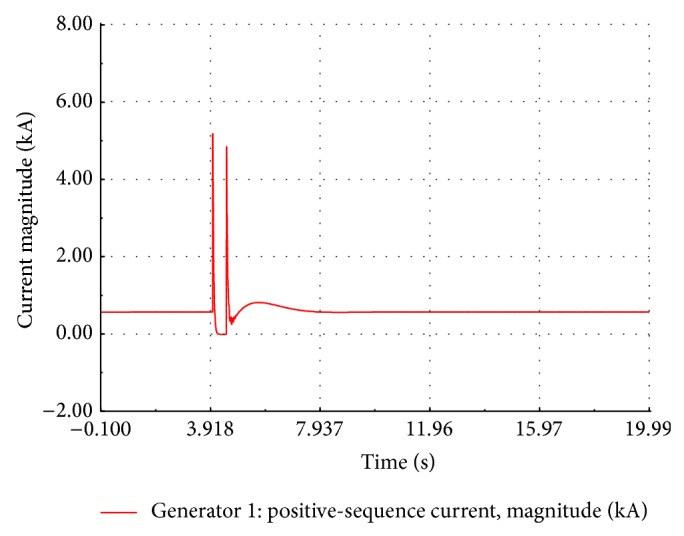
Simulated current of FSWEG1.

A three-phase symmetrical fault is created across the FSWEG1 bus at *t* = 4.0 s for duration of 500 ms. The simulation time is set to 20 seconds. During the fault period, the bus voltage of FSWEG1 drops to zero whereas the bus voltages of other FSWEGs decrease. After the fault clearance, the voltage of the FSWEG1 rapidly increases to 0.685 kV at *t* = 4.814 sec and then voltage undershoots to a value of 0.621 kV and finally reaches the steady state value as shown in Figure 12. The variation of the current during the fault period is as shown in Figure 13. It is clear from the graph that the RMS current increases to 5 times the nominal value at the instant of the fault and then, within 250 ms, the current decays to zero value. During the fault clearance, the generator transient current reaches the peak value of 3 kA and then settles to the nominal value of 0.6 kA within 3.4 seconds.

**Figure 14:**
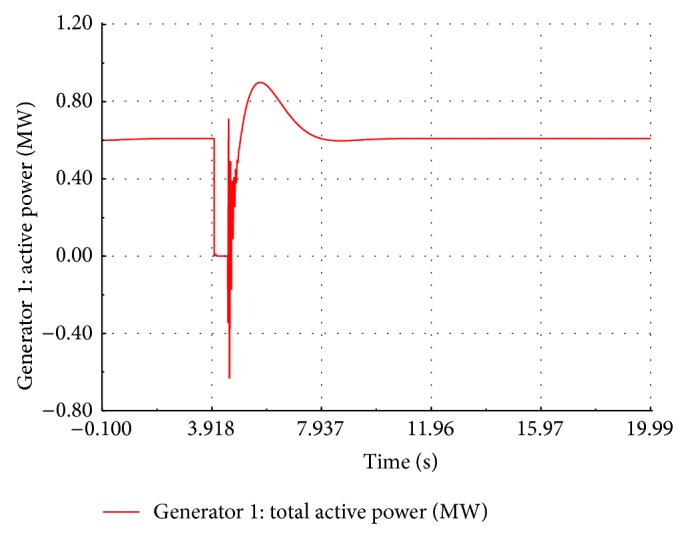
Simulated real power of FSWEG1.

**Figure 15:**
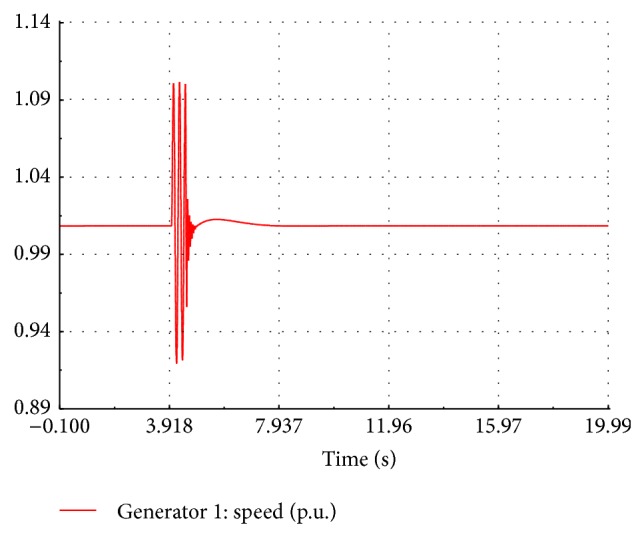
Simulated speed of FSWEG1.

At the instant of fault occurrence, the active power drops to zero and remains the same during the fault period without any transient as shown in Figure 14. After the fault is cleared, the response of active power is oscillatory with first peak being 1.8 times the nominal value and reaches the steady state at 3.2 seconds. During the fault, the speed response is oscillatory with the frequency of 5 Hz and a peak to peak value 0.22 p.u. as shown in Figure 15. After the fault is cleared, the speed response again becomes oscillatory with frequency of 10 Hz and a peak to peak value of 0.1 p.u. The speed finally settles down to its nominal value after 2.3 sec. During the fault, the performance of wind turbine is also affected. It is observed in the site that the speed of the wind turbine is increased above the rated speed.

### 5.3. Interruption of Wind Electric System

**Figure 16:**
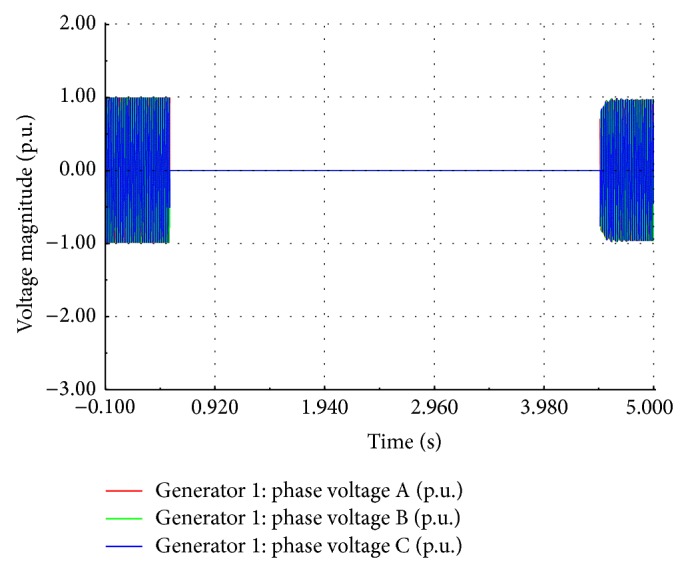
Simulated interrupted voltage waveform.

Interruption of wind electric generator is caused either by tripping of generator manually or by automatic tripping when the wind speed falls below the cut-in value. For the wind speed below 5 m/s, the induction generator works as motor and begins to absorb the real and reactive power from the grid. At this instant, the soft starter disconnects the wind generator from the grid leading to power interruption. The wind turbine initially accelerates at the rate of 14 rpm/sec if the generator is manually tripped while it is delivering the rated power. The speed increases to a dangerously high value, initiating the application of mechanical break to bring the turbine to a standstill position. The simulated interruption voltage waveform at the generator bus is shown in Figure 16 which matches well with the recorded waveform as shown in Figure 2(d).

### 5.4. Capacitor Switching

**Figure 17:**
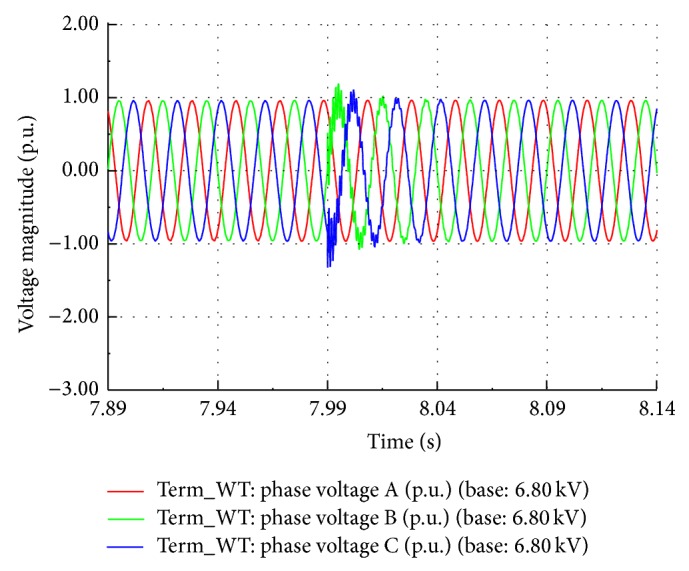
Simulated capacitor switching waveform.

A fixed capacitor bank of 1 MVAR is connected at 11 kV substation bus to provide no load reactive VAR compensation of about 30% of total wind power generation. When the capacitor bank is switched on to the bus, the generator busbar voltage increases to 1.44 p.u. and the current suddenly rises to 1.3 p.u. and finally settles down to the nominal value within 2 seconds. The EMT simulation is carried out to find the variations in the instantaneous phase voltages after the switching event. The simulated waveform is as shown in Figure 17 which closely matches the recorded waveform shown in Figure 2(a).

### 5.5. Voltage Sag

**Figure 18:**
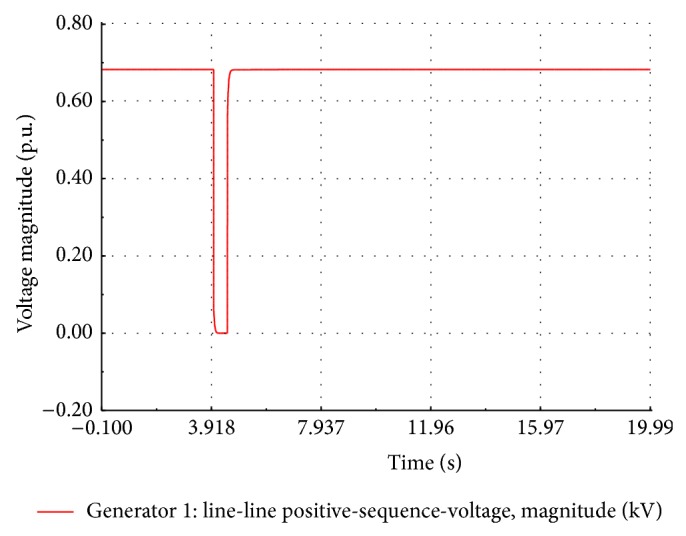
Simulated voltage sag in p.u.

**Figure 19:**
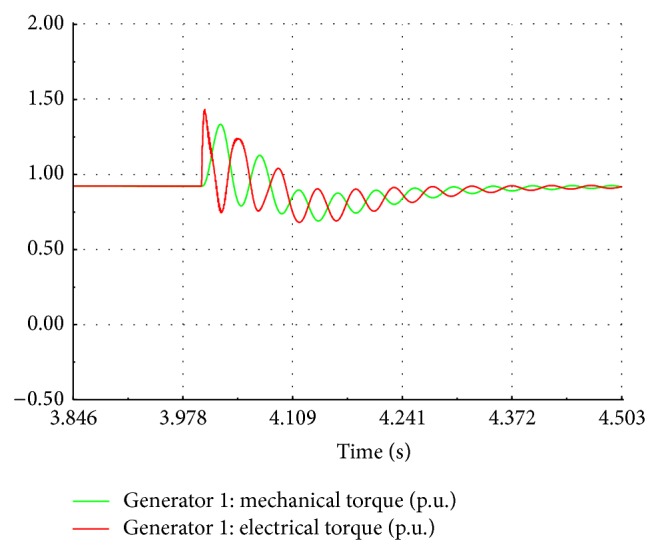
Electrical and mechanical torque.

**Figure 20:**
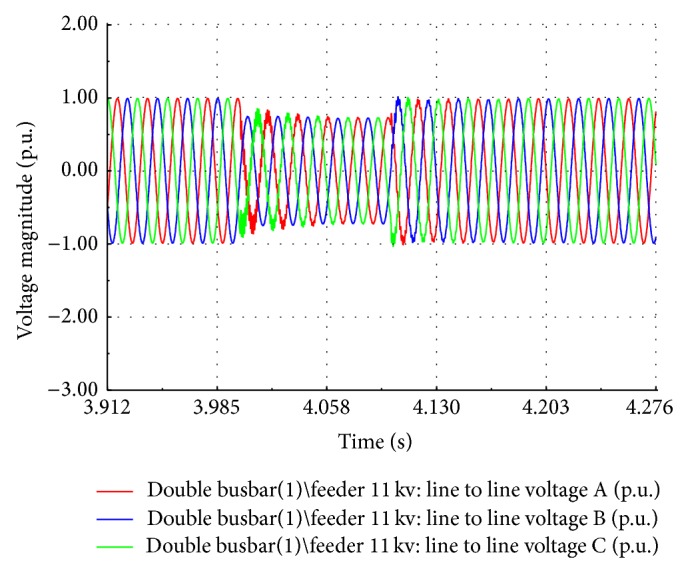
Substation bus 11 kV: line to line voltage in kV.

The voltage sags are the frequently recorded power quality events in the substation under study. Eleven events with time duration between 0.3 sec and 1 min and nine events with time duration between 1.1 min and 3 min are recorded as per EN50162 standard. The induction motor load is connected to the 11 kV busbar at *t* = 4 seconds during simulation. The sag event is observed during the simulation period of 10 seconds. The voltage at the transformer busbar drops to 0.88 p.u. for a duration of 0.7 seconds as shown in Figure 18. The dip in voltage depends on the short circuit ratio of the grid. For a weak grid, the percentage of voltage dip is high. The active power exported to the grid is reduced and the electromagnetic torque drops to 0.87 p.u. causing an increase in the rotor speed as shown in Figure 19.  Figure 20 shows the EMT simulation of instantaneous line voltage of generator bus during sag event and it matches well with the recorded waveform as shown in Figure 2(b).

### 5.6. Voltage Swell

Swell is an event where the voltage magnitude varies between 1.1 p.u. and 1.8 p.u. for an interval ranging from 0.5 to 1 minute. The swell can occur due to a single line to ground fault on the system or removal of unbalancing load causing temporary voltage rise on the unfaulted phases.

**Figure 21:**
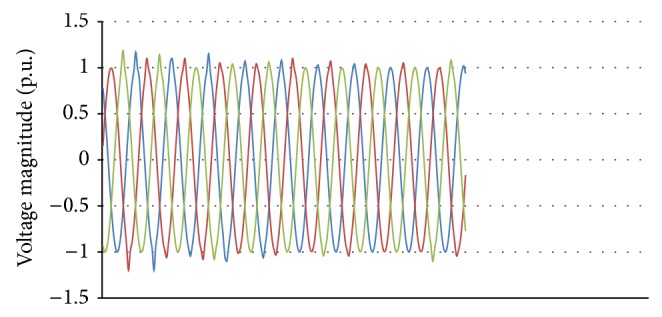
Waveform of simulated voltage swell.

Initially, the system is operating under normal load conditions and load is removed suddenly from the 11 kV busbar. At this stage, sharp rise in voltage is observed as depicted in Figure 21. These waveforms are similar to those recorded by the power quality analyzer in the substation as shown in Figure 2(c) which validates the simulation model proposed for FSWES.

## 6. Remedial Measure for Sag

From the analyses of the recorded power quality data, it is observed that the voltage impulse transients and sags are the major events in the substation. The effect of these events can be reduced by providing the most commonly used compensating device STATCOM connected at 11 kV substation bus. A three-phase symmetrical ground fault is applied to the generator_1 bus at *t* = 4.0 s for a duration of 500 ms for studying the voltage profile of busbar with and without STATCOM.

When the fault is cleared, the bus voltage of the generator_1 rapidly rises and settles down to nominal value of 0.69 kV within 0.1 seconds. The reactive power injected by the STATCOM increases to 3 times the nominal value of 2 MVAR for improving the voltage profile.

The low frequency oscillation of the generator speed is reduced during the fault period with STATCOM and the oscillation settles down within short span of 0.5 seconds after the fault clearance.  Table 2 shows the improvement of voltage profile at various locations in the substation.

## 7. Conclusion

In the proposed study, power quality related to the substation with large number of wind turbines located in Coimbatore region in Tamil Nadu state is considered for improving the wind power penetration into the grid. Power quality analyzers are installed at the selected wind turbine and group control feeders. The various data recorded by the instrument are analysed according to EN5160 standard. From the analysis it is clear that the major events are sags and impulsive transients.

With a view to studying the behavior of substation for different operating scenarios, a simulation model of the substation with fixed speed squirrel induction generator is developed using DIgSILENT software. In this model, pitch angle control is implemented to keep the power output as constant even when the wind speed is above the rated value up to cut-out speed. In order to keep the power at rated value for high wind speeds, the power coefficient *C*_*p*_ is varied based on the lookup table relating the wind speed, beta, and the tip speed ratio. The design is based on data taken from the turbine characteristic available at the substation. The dynamic interaction between the wind farm and the substation is studied from the simulation model for change in wind speed, short circuit, and sudden change in load, switching of capacitor, and tripping of wind turbine. The response and the waveform in the simulated model of the substation for various events closely match with those recorded events by the instrument, thus validating the simulation model.

## Figures and Tables

**Table 1:** Length of the cable in meter.

*L*1	*L*2	*L*3	*L*4	*L*5	*L*6

0.2	0.2	0.7	1	0.5	1.75

**Table 2:** Voltage profile.

Voltage in p.u.	Without STATCOM	With STATCOM
Generator_1 busbar voltage	0	0.2
Generator_6 busbar voltage	0.26	0.45
110 kV busbar voltage	0.31	0.534
